# Perioperative Safety and Efficacy of Holmium Laser Enucleation of the Prostate in Patients Receiving Antithrombotic Therapy: A Prospective Cohort Study

**DOI:** 10.1038/s41598-020-61940-0

**Published:** 2020-03-24

**Authors:** Hyeong Dong Yuk, Seung-June Oh

**Affiliations:** 1 Department of Urology, Seoul National University Hospital, Seoul, Republic of Korea; 2 Department of Urology, Seoul National University College of Medicine, Seoul National University Hospital, Seoul, Republic of Korea

**Keywords:** Prostate, Urological manifestations

## Abstract

We investigated the efficacy of and risk from holmium laser enucleation of the prostate (HoLEP) due to discontinuation of antithrombotics in patients with benign prostatic hyperplasia (BPH). Patients in the prospective SNUH-BPH Database Registry who underwent HoLEP between December 2010 and December 2017 were enrolled. Preoperative evaluation included symptom score questionnaires, laboratory tests, urine tests, prostate-specific antigens, urodynamic study, and transrectal ultrasonography. Postoperative evaluation was performed at 2 weeks, 3 months, and 6 months. Information regarding the types of antithrombotics and their use, underlying disease, and antithrombotic management during surgery was collected. The study included 55 patients. The mean age and preoperative prostate volume were 68.7 ± 6.4 years and 70.3 ± 32.2 mL, respectively. The mean preoperative hemoglobin level was 13.5 ± 2.6 g/dL in the patients receiving antithrombotics. Of the patients, 71% were taking aspirin. Seventy-five (66.5%) and 70 patients (28.2%) discontinued the antithrombotic therapy 5–7 days and <1 week preoperatively, respectively. Three patients (1.21%) were switched to low-molecular-weight heparin therapy, and 10 (4.03%) continued antithrombotic therapy. No significant differences were found in the incidence rates of postoperative transfusion (p = 0.894) or complications from antithrombotic use, thrombosis (p = 0.946), haemorrhage requiring bladder irrigation (p = 0.959), transurethral coagulation (p = 0.894), cardiovascular events (p = 0.845), and cerebrovascular events (p = 0.848). Efficacy and complications related to the short-term antithrombotic withdrawal before and after HoLEP also showed no significant differences. HoLEP may be a beneficial surgical technique for patients with BPH who are receiving antithrombotics.

## Introduction

Cardiovascular diseases (CVDs) include vascular and heart diseases, including atherosclerosis, peripheral vascular diseases, and cerebrovascular diseases. Cerebrovascular accidents (CVAs) include ischemic stroke, transient ischemic attack, and haemorrhagic stroke such as subarachnoid and intracerebral haemorrhages. CVDs are among the most common causes of adult death in the United States, with 1 in 3 adults dying each year^1^. CVA is the third leading cause of death in the United States, following heart disease and cancer^2^. The incidence rates of CVA and CVD increase with age^1^. Various treatments are available for patients with CVA and CVD, and those at risk, with the most common treatment being antithrombotic therapy^3^. As the CVA and CVD risk and incidence rates increase with age, the number of patients taking antithrombotics is also increasing; many of these patients have BPH that requires surgical treatment^4^. For this reason, urologists often perform BPH surgery in patients with CVA and CVD who are receiving antithrombotics^5^.

Benign prostatic hyperplasia (BPH) is one of the most common diseases in men aged >50 years, and its incidence increases with age >50 years^6^. For decades, the standard of surgical treatment for BPH was transurethral prostatectomy (TURP)^7^. However, TURP is known to be associated with high incidence rates of perioperative bleeding, transfusion, and delayed bleeding^8^. Bleeding-related problems can also occur, such as increased catheter maintenance duration, bladder irrigation time, and length of hospital stay, which can arise from TURP^8–10^. As an alternative to this problem, interests in minimally invasive surgery and less-invasive surgery have increased. The recent increasing interest in noninvasive surgery has led to the popularization of laser-assisted BPH surgery^11^. Holmium laser enucleation of the prostate (HoLEP) is one of the laser-assisted BPH surgeries.

Only few studies have included a large number of patients or have been conducted on Asian populations. The number of patients with risk factors of CVA or CVD is increasing because of the aging of society^1^. Therefore, we investigated the treatment efficacy and risks of CVA and CVD in patients who underwent HoLEP with antithrombotic therapy among Koreans.

## Methods

This study reviewed a prospective cohort of patients who underwent HoLEP with LUTS/BPH. The BPH prospective cohort is part of the previous prospective SNUH-BPH Database Registry, which was approved by the Institutional Review Board of Seoul National University Hospital (IRB No. H-0810-027-260). We obtained informed consent from the patients. Study protocol and contents associated with this study followed the Declaration of Helsinki guidelines.

The inclusion criteria included patients aged ≥50 years who had a clinical diagnosis of BPH and received HoLEP. The exclusion criteria were patients with a history of genitourinary cancer and pelvic surgery, and neurogenic bladder dysfunction. Digital rectal examination, serum prostate specific antigen (PSA) level, international prostate symptom score (IPSS), overactive bladder symptom score (OABSS), 72-h voiding diary, cystourethroscopy, transrectal ultrasonography (TRUS), laboratory serum tests, and urine tests were used for baseline evaluation. Most of the antibiotics used were second-generation cephalosporins or ciprofloxacin for patients with hypersensitivity reactions. HoLEP was performed in the lithotomy position under general or spinal anaesthesia. Enucleation was performed with the three-lobe or early inverted V-shaped mucosal incision with anterior conjoining techniques^12^ using a holmium:YAG laser (Lumenis, The VersaPulse PowerSuite 100 W) with a 550-mm end laser fibre (Boston Scientific, AccuMax 550 Laser Fiber) and a laser setting of 80 W (2J × 40 Hz). The enucleated prostatic adenoma was morcellated with a morcellator (Lumenis, Versacut). After the operation, bladder irrigation was performed using normal saline. The intraoperative parameters included operative time, energy use, weight of enucleated prostate tissue, morcellation time, and intraoperative complications such as bladder injury and prostate capsule perforation. All the patients who were treated with antithrombotic therapy were instructed to resume their antithrombotic medications when haematuria was no longer visible.

Information on the use of antithrombotics and discontinuation methods during operation was collected. Postoperative parameters such as IPSS, OABSS, 72-h voiding diary, uroflowmetry, urine test, and PSA were evaluated. In addition, data regarding complications such as UTI, urgency, urinary incontinence, postoperative bleeding (which required transurethral coagulation or bladder irrigation), postoperative urinary retention, re-urethral catheterization, urethral stricture, bladder neck contracture, and cerebrovascular and cardiovascular events were collected. Follow-up was performed at 2 weeks, 3 months, and 6 months postoperatively.

The patients were categorized into two groups, a non-antithrombotic therapy group and an antithrombotic therapy group. The various clinical parameters, and the postoperative outcomes and complications associated with the use of antithrombotics were compared between the two groups.

All statistical analyses were performed using IBM SPSS Statistics version 22.0 (IBM, Armonk, NY, USA). Continuous variables were expressed as median and interquartile range (IQR), or mean and standard deviation (SD). Categorical variables were expressed using the ratio of events (%). Quantitative data were analysed using the Student *t* test. Binary variables were analysed when a chi-square test was applicable, but the Fisher exact test was performed if the distribution of the equation deviated significantly from normal. Multivariate analysis was performed using logistic regression. All statistical analyses were two-sided, and statistical significance was defined as a p value of ≤0.05.

## Results

The data of 955 patients from a prospective database of patients who underwent HoLEP with BPH between December 2010 and December 2017 were analyzed. We found that 707 patients (74.0%) did not take antithrombotics before surgery and 248 (25.9%) took ≥1 antithrombotic before surgery. All the patients were followed up for 6 months. Table 1 summarizes the baseline characteristics of the patients. In both groups, the patients in the antithrombotic group were relatively older, had a higher body mass index, and had an underlying disease such as hypertension, diabetes, neurological disease, CVD, cerebrovascular disease, or chronic kidney disease. Both groups had normal hemoglobin levels, but the patients in the antithrombotic group had slightly lower hemoglobin levels.

**Table 1:** Baseline patient characteristics of non- antithrombotic and antithrombotic groups.

Variables	Non-antithrombotic (N = 707)	antithrombotic (N = 248)	P-value
Age (years)	68.4 ± 7.1	70.4 ± 6.4	<0.001
BMI (kg/m^2^)	23.9 ± 2.7	24.7 ± 3.2	0.001
Duration of LUTS (months)	56.3 ± 54.4	52.4 ± 46.9	0.284
Previous BPH operation	34 (4.8%)	17 (6.9%)	0.285
Preoperative Hb (g/dL)	14.0 ± 1.8	13.5 ± 2.6	0.010
**Medical treatment**			
Alpha blockers	449 (63.5%)	163 (65.7%)	0.583
Anticholinergics	27 (3.8%)	10 (4.0%)	0.996
5α-Reductase inhibitor	167 (23.6%)	64 (25.8%)	0.545
Desmopressin	15 (2.1%)	5 (2.0%)	0.954
**Comorbidities**			
Diabetes	84 (11.9%)	91 (36.7%)	<0.001
Hypertension	233 (33.0%)	163 (65.7%)	<0.001
Cardiovascular diseases	23 (3.3%)	59 (23.8%)	<0.001
Chronic kidney disease	6 (0.8%)	10 (4.0%)	<0.001
Cerebrovascular disease	12 (1.6%)	38 (15.4%)	<0.001
Neurologic disease	57 (8.1%)	46 (18.5%)	<0.001

BMI: body mass index; LUTS: lower urinary tract symptoms.

Next, Table 2 summarizes the antithrombotic medications, indications or causes, and the treatment during surgery for patients in the antithrombotic group. Aspirin was the most commonly used drug, accounting for 71% of cases, followed by clopidogrel (22.9%). All other drugs were used in <3% of the patients. The most common cause of antithrombotic use was angina, accounting for 30% of the antithrombotic users. The next two leading causes of antithrombotic use were artery disease prevention (10.1%) and cerebrovascular disease (8.9%). During the operation, 95.7% of the patients discontinued antithrombotics, while 5.3% of the patients replaced the antithrombotic with LMWH or maintained the original antithrombotic.

**Table 2:** Type of oral antithrombic drug, indication and management during the operation of antithrombic group.

	Antithrombic (N = 248)
***Type of antithrombic drug***	
Aspirin	176 (71.0%)
Clopidogrel	57 (22.9%)
Cilostazol	6 (2.4%)
Warfarin	3 (1.2%)
Sarpogrelate	3 (1.2%)
Apixaban	2 (0.8%)
Dabigatran	1 (0.4%)
Ticlopidine	1 (0.4%)
Rivaroxaban	1 (0.4%)
Combination of antithrombotic drug	17 (1.7%)
***Indications for oral antithrombic therapy***	
Angina	77 (31%)
Prevention of arterial disease	25 (10.1%)
Cerebrovascular disease	22 (8.9%)
Chronic atrial fibrillation	15 (6.0%)
Myocardial infarction	14 (5.6%)
Aortic aneurysm	4 (1.6%)
Congestive heart failure	2 (0.8%)
Pulmonary embolism	1 (0.4%)
***Management during the operation***	
Stop for 7 days	165 (66.5%)
Stop for 3–5 days	70 (28.2%)
Replaced with LMWH	3 (1.2%)
Antithrombotic maintained	10 (4.1%)

LMWH: low molecular weight heparin.

Table 3 compares the perioperative outcomes between the two groups. No significant differences in prostate volume and mean maximum urinary flow (Qmax) were found. The intraoperative parameters such as operation time, removed prostate weight, and complications were also not significantly different between the two groups. The immediate postoperative parameters showed no significant differences, particularly the number and duration of the additional continuous bladder irrigations and hospitalization duration. However, we found a significant difference in the duration of Foley catheter retention (p = 0.017), but the difference from that in clinical practice was not significant.

**Table 3:** Perioperative outcomes of non-antithrombic and antithrombic groups.

Variables	Non- antithrombic (N = 707)	Antithrombic (N = 248)	P-value
***Preoperative parameters***			
**Prostate volume (mL)**			
Total volume	69.3 ± 35.8	71.7 ± 36.3	0.369
Transition zone volume	41.1 ± 27.5	41.5 ± 25.4	0.815
Qmax (ml/s)	7.1 ± 4.5	7.7 ± 3.8	0.052
***Intraoperative parameters***			
Morcellation time (min)	10.3 ± 9.4	9.6 ± 6.3	0.233
Total operation time (min)	57.0 ± 30.7	55.2 ± 26.5	0.395
Enucleation weight (g)	23.5 ± 21.8	23.2 ± 19.1	0.844
Energy used (KJ)	80.5 ± 49.5	81.7 ± 34.9	0.671
Intraoperative bladder injury	6 (0.8%)	2 (0.8%)	0.999
Interoperative capsule perforation	4 (0.6%)	2 (0.8%)	0.959
Intraoperative bleeding event	25 (3.5%)	10 (4.0%)	0.872
***Immediate postoperative parameters***			
Postoperative urethral catheter duration (day)	1.7 ± 2.2	1.4 ± 1.5	0.017
Additional continuous bladder irrigation	28 (4.0%)	8 (3.2%)	0.742
Duration of additional CBI			0.094
1 day	24 (3.4%)	5 (2.0%)	
2 days	0 (0.0%)	2 (0.8%)	
≥ 3 days	3 (0.4%)	0 (0.0%)	
Hospitalization			0.351
1 day	661 (93.5%)	236 (95.2%)	
2 days	40 (5.7%)	10 (4.0%)	
≥ 3 days	6 (0.8%)	2 (0.8%)	

CBI: continuous bladder irrigation; Qmax: the mean maximum urinary flow.

Furthermore, Tables 4 and 5 compares the incidence of postoperative complications between the two groups at 2 weeks, 3 months, and 6 months. Transfusion within 2 weeks after operation occurred in one case for both groups. No significant differences were found between the two groups in terms of the incidence of complications related to blood clot and bleeding requiring transurethral coagulation (TUC) or continuous bladder irrigation. In addition, no significant differences in the incidence of complications were found between 3 and 6 months after operation. However, among the patients who temporarily discontinued antithrombotic therapy, one (0.4%) had an intracerebral infarction and another (0.4%) had a myocardial infarction.

**Table 4:** Postoperative complications of non-antithrombic and antithrombic groups.

Variable	Non- antithrombic (N = 707)	Antithrombic (N = 248)	P-value
***Postoperative 2 week***			
Urinary incontinence	112 (15.8%)	50 (20.2%)	0.144
Urgency	142 (20.1%)	42 (16.9%)	0.323
Transfusion	1 (0.1%)	1 (0.4%)	0.894
Recatheterization	38 (5.4%)	15 (6.0%)	0.812
Clot related problem	25 (3.5%)	9 (3.6%)	0.946
Urinary retention	21 (3.0%)	8 (3.2%)	0.951
Bleeding need for CBI	14 (2.0%)	5 (2.0%)	0.959
Bleeding need for TUC	1 (0.1%)	1 (0.4%)	0.894
Urinary tract infection	7 (1.0%)	2 (0.8%)	0.815
Cardiovascular event	0 (0%)	1 (0.4%)	0.845
Cerebrovascular event	0 (0%)	1 (0.4%)	0.848
***Postoperative 3 months***			
Stress urinary incontinence	36 (5.1%)	22 (8.9%)	0.047
Urgency urinary incontinence	46 (6.5%)	28 (11.3%)	0.022
Urgency	27 (3.8%)	14 (5.6%)	0.299
Urethral stricture	4 (0.6%)	2 (0.8%)	0.866
Bladder neck contracture	4 (0.6%)	0 (0.0%)	0.538
Urinary tract infection	8 (1.1%)	3 (1.2%)	0.915
Cardiovascular event	0 (0%)	0 (0%)	1.000
Cerebrovascular event	0 (0%)	0 (0%)	1.000
***Postoperative 6 months***			
Stress urinary incontinence	14 (2.0%)	10 (4.0%)	0.123
Urgency urinary incontinence	20 (2.8%)	11 (4.4%)	0.308
Urgency	15 (2.1%)	7 (2.8%)	0.699
Urethral stricture	1 (0.1%)	3 (1.2%)	0.095
Bladder neck contracture	6 (0.8%)	1 (0.4%)	0.783
Urinary tract infection	1 (0.1%)	1 (0.4%)	0.653
Cardiovascular event	0 (0%)	1 (0.4%)	0.849
Cerebrovascular event	0 (0%)	1 (0.4%)	0.856

CBI: continuous bladder irrigation; TUC: transurethral coagulation.

**Table 5:** Clavien-Dindo classification of non-antithrombic and antithrombic groups.

Variable	Non-antithrombic (N = 707)	Antithrombic (N = 248)	P-value
***Postoperative 2 week***			
Grade 1	248 (35.1%)	99 (39.9%)	0.198
Grade 2	15 (2.1%)	6 (2.4%)	0.981
Grade ≥3	15 (2.1%)	7 (2.8%)	0.699
***Postoperative 3 months***			
Grade 1	107 (15.1%)	44 (17.7%)	0.386
Grade 2	46 (6.5%)	28 (11.3%)	0.022
Grade ≥3	8 (1.1%)	2 (0.8%)	0.944
***Postoperative 6 months***			
Grade 1	90 (12.7%)	44 (17.7%)	0.064
Grade 2	23 (3.3%)	8 (3.2%)	0.948
Grade ≥3	9 (1.3%)	3 (1.2%)	0.954

The voiding parameters and symptom scores were not significantly different between the two groups before and after HoLEP. In both groups, the postoperative Qmax was increased and PVR was decreased significantly when compared with their preoperative values. After operation, positive changes were observed. No significant differences in the preoperative, 2-week, 3-month, and 6-month postoperative values of Qmax and PVR were found between the two groups. In the symptom scores, the IPSS improved in both groups as compared with the preoperative score, and no significant difference was found between the two groups.

## Discussion

Haematuria is the most common complication of surgery for BPH. In some cases, transfusions may be necessary owing to persistent or late postoperative bleeding. These complications are related to the preoperative hypercoagulability state and present in many patients taking antithrombotics or coumarin derivatives relatively. Recently, aging and Western eating habits have increased the number of patients with thromboembolic diseases^13^. Many of these patients need and receive oral antithrombotic treatment^13^. However, prospective studies are lacking in patients who have been receiving long-term oral antithrombotic therapy prior to HoLEP.

Hochreiter *et al*. reported the benefits of HoLEP for patients taking antithrombotics first. In the study, 19 patients taking antithrombotics had undergone HoLEP and did not receive postoperative transfusion^14^. Bolton *et al*. reported that in a TURP study with coumarin-treated patients, 8% of the patients had long-term haematuria, 6% needed transfusion, and 2% required reoperation for haemostasis^15^. Descazeaud *et al*. reported their experience with 83 patients with high-risk bleeding tendency in a HoLEP study of patients taking oral antithrombotics. Of the 83 patients, 81 were taking oral antithrombotics and 2 had haemophilia. Of these patients, 33 discontinued taking oral antithrombotics before surgery, 34 shifted to low-molecular-weight heparin (LMWH), and 14 continued oral antithrombotic during operation. Transfusion was performed in 7 patients who discontinued oral antithrombotics, 5 with a LMWH replacement, and 7 who continued antithrombotic therapy^4^. In another study of 81 patients who underwent HoLEP, 14 patients maintained oral antithrombotics during surgery, but the transfusion rate was not significantly high^16^. When taking oral antithrombotics, HoLEP was recommended as a more suitable operation than TURP^16^. In the present study, 235 patients (95.7%) discontinued antithrombotic therapy. Three patients (1.2%) shifted to LMWH and 10 (4.1%) continued the use of the original antithrombotic. Transfusion was performed in 1 patient with LMWH replacement.

Marchion *et al*. reported acute cardiovascular events in 18 patients (1.9%) who underwent GreenLight laser photoselective vaporization among 923 patients who were taking antithrombotics. The distribution of cardiovascular events was as follows: 4 patients with myocardial infarction, 7 with angina pectoris, 3 with venous thromboses, 1 with atrial fibrillation, 1 with hypotension, 1 with a vasovagal reflex episode, and 1 with supraventricular tachycardia^17^. In their study of 305 patients receiving antithrombotics before TURP, Raj *et al*. reported that cardiovascular and cerebrovascular events occurred at incidence rates of 0.98% and 0.65%, respectively^18^. Taylor *et al*. reported the occurrence of cardiovascular complications in 6 (9.2%) of 65 patients who discontinued antithrombotics before and after surgery^19^.

In the present study, the incidence of clot-related problem was 3.6%, the retention rate was 3.2%, the frequency of continuous bladder irrigation due to postoperative bladder bleeding was 2%, and the frequency of TUC in the antithrombotic group was 0.4%. The mean catheterization period was 1.4 days, and the mean length of hospital stay was 1 day. Of the patients, 95.2% were hospitalized for 1 day and only 4.8% were hospitalized for >1 day. Furthermore, among the patients who temporarily discontinued antithrombotic therapy, one (0.4%) had an intracerebral infarction and another (0.4%) had a myocardial infarction. No significant difference was found in the incidence of postoperative transfusion (p = 0.884) or complication with antithrombotic administration. Moreover, no significant difference was found in the incidence of haemorrhage (p = 0.959, p = 0.894) requiring thrombosis (p = 0.946), bladder washing, and transurethral coagulation. We found no significant differences in the incidence of complications between 2 weeks, 3 months, and 6 months after surgery.

Holmium laser does not penetrate deeply into the tissue, penetrating only at a depth of 0.4 mm. It achieves rapid vaporization and solidification of the tissue, and this is beneficial for regulating haemostasis in patients taking antithrombotics^20^.

This study analysed prospectively collected data, with an attempt to minimize bias. However, it still has limitations. The study cohort was followed up for 6 months, with limited results for long-term outcomes or complications occurring after 6 months. Future studies could examine patients several years postoperatively to provide better understanding and stronger implications regarding the efficacy of HoLEP as a surgical method for patients with BPH who are using antithrombotics in the long term.

## Conclusion

This study suggests that HoLEP is an effective surgical method for patients with BPH who are taking antithrombotics. We observed a few cardiovascular and cerebrovascular complications related to short-term antithrombotic withdrawal before and after HoLEP in the patients with BPH who were receiving antithrombotic therapy.
